# Does the Ranking Matter? A Retrospective Cohort Study Investigating the Impact of the *2018 CANMAT and ISBD Guidelines for the Management of Patients with Bipolar Disorder* Treatment Recommendations for Acute Mania on Rehospitalization Rates

**DOI:** 10.1177/07067437231156235

**Published:** 2023-02-21

**Authors:** Fabiano A. Gomes, Henrique Dumay, Julia Fagen, Natalie Palma, Roumen Milev, Elisa Brietzke

**Affiliations:** 1 Department of Psychiatry, 4257 Queen's University, Kingston, ON, Canada; 2 Centre for Neuroscience Studies, 4257 Queen's University, Kingston, ON, Canada; 3 Department of Psychiatry and Behavioural Neurosciences, 3710 McMaster University, Hamilton, ON, Canada; 4 28127 University of Brasilia, Brasilia, Brazil; 5 Providence Care Hospital, Kingston, ON, Canada

**Keywords:** bipolar disorder, mania, rehospitalization, clinical guidelines, CANMAT, ISBD

## Abstract

**Objective:**

There is limited data about the impact of mood disorders treatment guidelines on clinical outcomes. The objective of this study was to investigate the impact of prescribers’ adherence to the 2018 Canadian Network for Mood and Anxiety Treatments (CANMAT) and International Society for Bipolar Disorders (ISBD) treatment guidelines recommendations on the readmission rates of patients hospitalized for mania.

**Method:**

A retrospective cohort of all individuals admitted due to acute mania to Kingston General Hospital, Kingston, ON, from January 2018 to July 2021 was included in this study. Patient variables and data regarding index admission and subsequent hospitalizations were extracted from medical records up to December 31, 2021. Treatment regimens were classified as first-line, second-line, noncompliant, or no treatment. We explored the associations between treatment regimens and the risk of readmissions using univariate, multivariate, and survival analysis.

**Results:**

We identified 211 hospitalizations related to 165 patients. The mean time-to-readmission was 211.8 days (standard deviation [SD]  =  247.1); the 30-day rehospitalization rate was 13.7%, and any rehospitalization rate was 40.3%. Compared to no treatment, only first-line treatments were associated with a statistically significant decreased risk of 30-day readmission (odds ratio [OR] = 0.209; 95% CI, 0.058 to 0.670). The risk of any readmission was reduced by first-line (OR = 0.387; 95% CI, 0.173 to 0.848) and noncompliant regimens (OR = 0.414; 95% CI, 0.174 to 0.982) compared to no treatment. On survival analysis, no treatment group was associated with shorter time-to-readmission (log-rank test, *p*  =  0.014) and increased risk of readmission (hazard ratio = 2.27; 95% CI, 1.30 to 3.96) when compared to first-line medications.

**Conclusions:**

Treatment with first-line medications was associated with lower 30-day rehospitalization rates and longer time-to-readmission. Physicians’ adherence to treatments with higher-ranked evidence for efficacy, safety, and tolerability may improve bipolar disorder outcomes.

## Abstract (fr)

**Objectif:**

Il y a des données limitées au sujet de l‘effet des lignes directrices du traitement des troubles de l’humeur sur les résultats cliniques. La présente étude avait comme objectif d’investiguer l’effet de l’adhésion des prescripteurs aux recommandations des lignes directrices de traitement 2018 CANMAT et ISBD concernant les taux de réhospitalisation des patients hospitalisés pour manie.

**Méthode:**

Une cohorte rétrospective de toutes les personnes hospitalisées en raison de manie aiguë à l’Hôpital général de Kingston, ON, de janvier 2018 à juillet 2021 était incluse dans cette étude. Les variables et données des patients concernant la première hospitalisation et les subséquentes ont été extraites des dossiers médicaux jusqu’au 31 décembre 2021. Les régimes de traitement étaient classés ainsi : première ligne, deuxième ligne, non-conforme, ou aucun traitement. Nous avons exploré les associations entre les régimes de traitement et le risque de réhospitalisations à l’aide d’analyse univariée, multivariée et de survie.

**Résultats:**

Nous avons identifié 211 hospitalisations liées à 165 patients. Le temps moyen jusqu’à la réhospitalisation était de 211,8 jours (ET  =  247,1); le taux de réhospitalisation de 30 jours était de 13,7 %, et le taux de toute réhospitalisation était de 40,3 %. Comparé à aucun traitement, seuls les traitements de première ligne étaient associés à un risque décru de réhospitalisation de 30 jours (RC 0,209; IC 0,058 à 0,670). Le risque de toute réhospitalisation était réduit par un régime de première ligne (RC 0,387; IC 0,173 à 0,848) et les régimes non conformes (RC 0,414; IC 0,174 à 0,982) comparé à aucun traitement. Dans l’analyse de survie, aucun groupe de traitements n’était associé à un temps plus court jusqu’à la réhospitalisation (test logarithmique par rang, p  =  0,014) et à un risque accru de réhospitalisation (rapport de risques 2,27; IC 1,30 à 3,96) quand ils étaient comparés aux médicaments de première ligne.

**Conclusions:**

Le traitement des médicaments de première ligne était associé à des taux plus faibles de réhospitalisation de 30 jours et à des temps plus longs avant la réhospitalisation. L’adhésion des médecins aux traitements indiquant un taux plus élevé d’efficacité, d’innocuité et de tolérabilité peut améliorer les résultats du trouble bipolaire.

## Introduction

Bipolar disorder (BD) treatment is complex and may pose significant challenges to clinicians, patients, and families.^1^ Mood episodes are highly recurrent and associated with severe impairment, morbidity, and mortality.^2^ Patients with mania or bipolar depression that require hospitalization are particularly vulnerable to relapses^3^ and their management focuses not only on short-term symptomatic improvement but also on preventive measures to decrease the risk of readmissions.^4^

Clinical practice guidelines are systematically developed statements to assist practitioner and patient decisions about appropriate healthcare for specific clinical circumstances.^5^ Several organizations have published mood disorders treatment guidelines designed to summarize the scientific literature about available interventions to reduce symptoms and achieve remission, prevent relapses, and improve functional impairment and quality of life.^6,7^ The Canadian Network for Mood and Anxiety Treatments (CANMAT) has joined forces with the International Society for Bipolar Disorders (ISBD) and has published three editions of a common guideline^8–10^ providing evidence-based recommendations for the management of BD. The 2018 CANMAT/ISBD guideline ranks interventions as first-, second-, and third-line as well as nonrecommended treatments based on levels of evidence for efficacy, clinical experience, and safety and tolerability issues.^10^

Despite the increasing number of available guidelines, there is limited evidence about its adoption in clinical practice,^11,12^ concordance with the recommendations,^13,14^ and improvement in treatment outcomes.^15,16^ This study aimed to investigate the effect of the *CANMAT and ISBD 2018 Guidelines for the Management of Patients with Bipolar Disorder* classification of treatment recommendations on readmission rates of BD patients hospitalized for acute mania.

## Patients and Methods

### Population

We conducted a retrospective cohort study including all individuals between 18 and 65 years old admitted due to acute mania to the inpatient unit of Kingston General Hospital (KGH), Kingston, ON, from January 2018 to July 2021. KGH is a tertiary hospital, part of the Kingston Health Science Centre, that provides acute psychiatric inpatient treatment for the Kingston, Frontenac, and Lennox & Addington region serving almost 500,000 residents in Southeastern Ontario.

Patients were initially identified by their discharge diagnosis and the admission for a manic episode was confirmed by a comprehensive chart review. Although the presence of psychotic features was not an exclusion criterion, individuals with a lifetime diagnosis of schizophrenia or schizoaffective disorder and with episodes better characterized as substance-induced psychosis were not included in the study, as patients with manic episodes secondary to medical conditions. Patients with multiple admissions were included in the study and each admission for a manic episode was included as an event.

### Outcome Measures

The primary outcome was inpatient psychiatric readmission within 30 days from discharge. Secondary outcomes were any inpatient psychiatric hospitalization and time to rehospitalization. The follow-up period for each patient was determined by the time of their hospital discharge until readmission, or until December 31, 2021, if it did not occur.

### Treatment Regimens

We classified the pharmacological regimens received during hospital admission into 3 groups (first-line, second-line, and noncompliant treatments) according to the *CANMAT and ISBD 2018 Guidelines for the Management of Patients with Bipolar Disorder*.^10^ First-line treatments included lithium, divalproex, quetiapine (alone or in combination with lithium or divalproex), asenapine (alone or in combination with lithium or divalproex), aripiprazole (alone or in combination with lithium or divalproex), risperidone (alone or in combination with lithium or divalproex), paliperidone, and cariprazine. Patients treated with long-acting injectable (LAI) formulations of aripiprazole and paliperidone were also classified as receiving first-line treatments. Second-line treatments included olanzapine (alone or in combination with lithium or divalproex), carbamazepine, lithium in combination with divalproex, ziprasidone, haloperidol, or electroconvulsive therapy (ECT). Patients were classified as receiving “noncompliant treatments” if treated with drugs not listed as first- or second-line medications (e.g., lurasidone, loxapine, and zuclopenthixol) or with a combination of antipsychotic medications in therapeutic doses (antipsychotic polypharmacy). Patients who required *pro re nata* (PRN) use of quetiapine, olanzapine, or loxapine for episodes of agitation were classified according to their main treatment. Patients who refused or did not receive pharmacological treatment or ECT were classified as having “no treatment.”

### Confounding Factors

To control for potential confounders, demographic information, and clinical variables were extracted from the charts and included age, gender, marital status, being on long-term disability (LTD) support, body mass index (BMI), presence of general medical comorbidities, personal and family history of psychiatric disorders, age of onset (defined as the age of the first mood episode), history of previous suicide attempts, length of stay during the index admission and status at discharge (if the patient left against medical advice or if they were discharged by the team).

### Statistical Analysis

We explored the associations between demographic, clinical variables and treatment regimens, and risk of readmissions using univariate analyses (χ^2^ and Fisher's exact test). Time to readmission was evaluated using a right-censored Kaplan–Meier survival analysis of readmissions according to treatment classification. Log-rank tests were conducted to compare overall and pairwise survival curves among different treatments. Univariate and multivariate Cox proportional hazards regressions were conducted to quantify risk and control for covariates that were statistically different between groups in the univariate analysis. All statistical tests were conducted using RStudio version 2022.07.1 and statistical significance was set at 0.05.

## Results

### Study Sample and Readmission Rates

We identified 211 hospitalizations for acute mania related to 165 patients. The 30-day readmission rate was 13.7% and any readmission rate was 40.3% during the study period, with no evidence of seasonal influence on admissions. The mean time to rehospitalization was 211.8 days (standard deviation [SD]  =  247.1 days). Table 1 presents the demographic and clinical characteristics of the sample according to their readmission status and Table 2 shows the frequency of different agents prescribed for the treatment of mania. There were no patients treated with third-line regimens in our sample.

**Table 1.** Demographic and Clinical Characteristics of the Sample According to Their Readmission Status.

		Readmission 30 days			Any readmission		
		Yes (%)	No (%)	*p*-value	Yes (%)	No (%)	*p*-value
Number of events		29 (13.7)	182 (86.3)		85 (40.3)	126 (59.7)	
Age, years (SD)		43.1 (14.9)	40.0 (14.6)	0.74	40.3 (13.5)	40.5 (15.4)	0.98
Gender	Male	14 (48.2)	86 (47.3)		40 (47.1)	60 (47.6)	
	Female	15 (51.8)	93 (51.1)	1	44 (51.8)	64 (50.8)	1
	Other	0 (0.0)	3 (1.6)		1 (1.1)	2 (1.6)	
Marital status	Married^a^	8 (27.6)	50 (27.5)	1	21 (24.7)	37 (29.4)	1
	Other	21 (72.4)	132 (72.5)		64 (75.3)	89 (70.6)	
Long-term disability^b^	Yes	18 (62.1)	57 (32.0)	0.003	44 (53.0)	31 (25.0)	<0.01
	No	11 (37.9)	121 (68.0)		39 (47.0)	93 (75.0)	
BMI, kg/m² (SD)		24.1 (6.8)	25.3 (5.7)	0.87	24.3 (5.9)	25.7 (5.9)	0.85
Family history of psychiatric disorders^c^	Yes	13 (46.4)	88 (52.7)	0.68	43 (51.8)	58 (51.8)	1
	No	15 (53.6)	79 (47.3)		40 (48.2)	54 (48.2)	
General comorbidities	Yes	23 (79.3)	114 (62.6)	0.12	61 (71.8)	76 (60.3)	0.12
	No	6 (20.7)	68 (37.4)		24 (28.2)	50 (39.7)	
Psychiatric comorbidities	Yes	21 (72.4)	159 (87.4)	0.05 (f)	73 (85.9)	107 (84.9)	1
	No	8 (27.6)	23 (12.6)		12 (14.1)	19 (15.1)	
Age of onset, years (SD)		21.8 (5.7)	29.4 (11.9)	0.28	25.0 (8.0)	30.4 (12.7)	0.47
Previous suicide attempts^d^	Yes	7 (28.0)	33 (20.2)	0.54	17 (22.4)	23 (20.5)	0.90
	No	18 (72.0)	130 (79.8)		59 (77.6)	89 (79.5)	
Length of hospital stay (index admission), days (SD)		22.5 (24.4)	18.1 (18.4)	0.49	21.3 (23.1)	17.0 (16.2)	0.49
Discharge type	AMA	6 (20.7)	19 (10.4)	0.12 (f)	14 (16.5)	11 (8.7)	0.14
	Discharged	23 (79.3)	163 (89.6)		71 (83.5)	115 (91.3)	
Treatment classification	First-line	5 (17.2)	79 (43.4)	0.04	29 (34.1)	55 (43.6)	0.09
	Second-line	7 (24.1)	32 (17.6)		16 (18.8)	23 (18.3)	
	Noncompliant	8 (27.6)	42 (23.1)		18 (21.2)	32 (25.4)	
	No treatment	9 (31.1)	29 (15.9)		22 (25.9)	16 (12.7)	
LAI medication	Yes	3 (10.3)	38 (20.9)	0.28	19 (22.4)	22 (17.5)	0.48
	No	26 (89.7)	144 (79.1)		66 (77.6)	104 (82.5)	

*Note*. BMI = body mass index; LAI = long-acting injectable; SD = standard deviation; AMA = against medical advice; (f) = Fisher exact test. ^a^Married also includes a common-law and stable partner. ^b^Data unavailable for 4 patients. ^c^Data unavailable for 16 patients. ^d^Data unavailable for 23 patients.

**Table 2.** Frequencies of Agents Prescribed for the Treatment of Mania Classified According to the 2018 CANMAT and ISBD Guidelines.

Classification	Agent	*n* (%)
First-line		84 (39.8)
	Lithium	04 (1.9)
	Divalproex	02 (0.9)
	Quetiapine	12 (5.7)
	Asenapine	02 (0.9)
	Aripiprazole (including LAI)	16 (7.6)
	Paliperidone (including LAI)	21 (10.0)
	Risperidone	—
	Cariprazine	—
	LI/DVP + quetiapine	16 (7.6)
	Li/DVP + aripiprazole (including LAI)	7 (3.3)
	Li/DVP + risperidone	3 (1.4)
	Li/DVP + asenapine	1 (0.5)
Second-line		39 (18.5)
	Olanzapine	16 (7.6)
	Carbamazepine	—
	Olanzapine + Li/DVP	23 (10.9)
	Lithium + DVP	—
	Ziprasidone	—
	Haloperidol	—
	ECT	—
Noncompliant		50 (23.7)
	Antipsychotic polypharmacy (including LAI)	25 (11.8)
	Li/DVP + antipsychotic polypharmacy (including LAI)	15 (7.1)
	Lurasidone	5 (2.4)
	Li/DVP + lurasidone	5 (2.4)
No treatment		38 (18.0)
Total		211 (100)

*Note*. CANMAT = Canadian Network for Mood and Anxiety Treatments; ISBD = International Society for Bipolar Disorders; LAI = long-acting injectable; Li = lithium; DVP = divalproex; ECT = electroconvulsive therapy.

There was no statistically significant difference between groups in most demographic and clinical variables except being financially supported by LTD (30-day and any readmission) and the prevalence of psychiatric comorbidities (30-day readmission). However, the overall prevalence of comorbidities was high (88.3%) and it was not associated with an increased risk of short-term readmission (OR=2.618; 95% CI, 0.896 to 7.093). Interestingly, there were no differences in baseline variables when participants were compared according to treatment regimens (Supplemental Table 1).

Patients who received any pharmacological treatment had lower rates of rehospitalizations when compared to patients who refused treatment but only treatment with first-line regimens was associated with a statistically significant decreased risk of 30-day readmission (OR=0.209; 95% CI, 0.058 to 0.670). Additionally, the risk of any readmission during the follow-up period was reduced by first-line (OR 0.387; 95% CI, 0.173 to 0.848) and noncompliant regimens (OR=0.414; 95% CI, 0.174 to 0.982) when compared to no treatment. There were no differences in readmission rates between patients treated with oral medications or LAI antipsychotics.

### Time to Readmission and Survival Analysis

**Figure 1.**
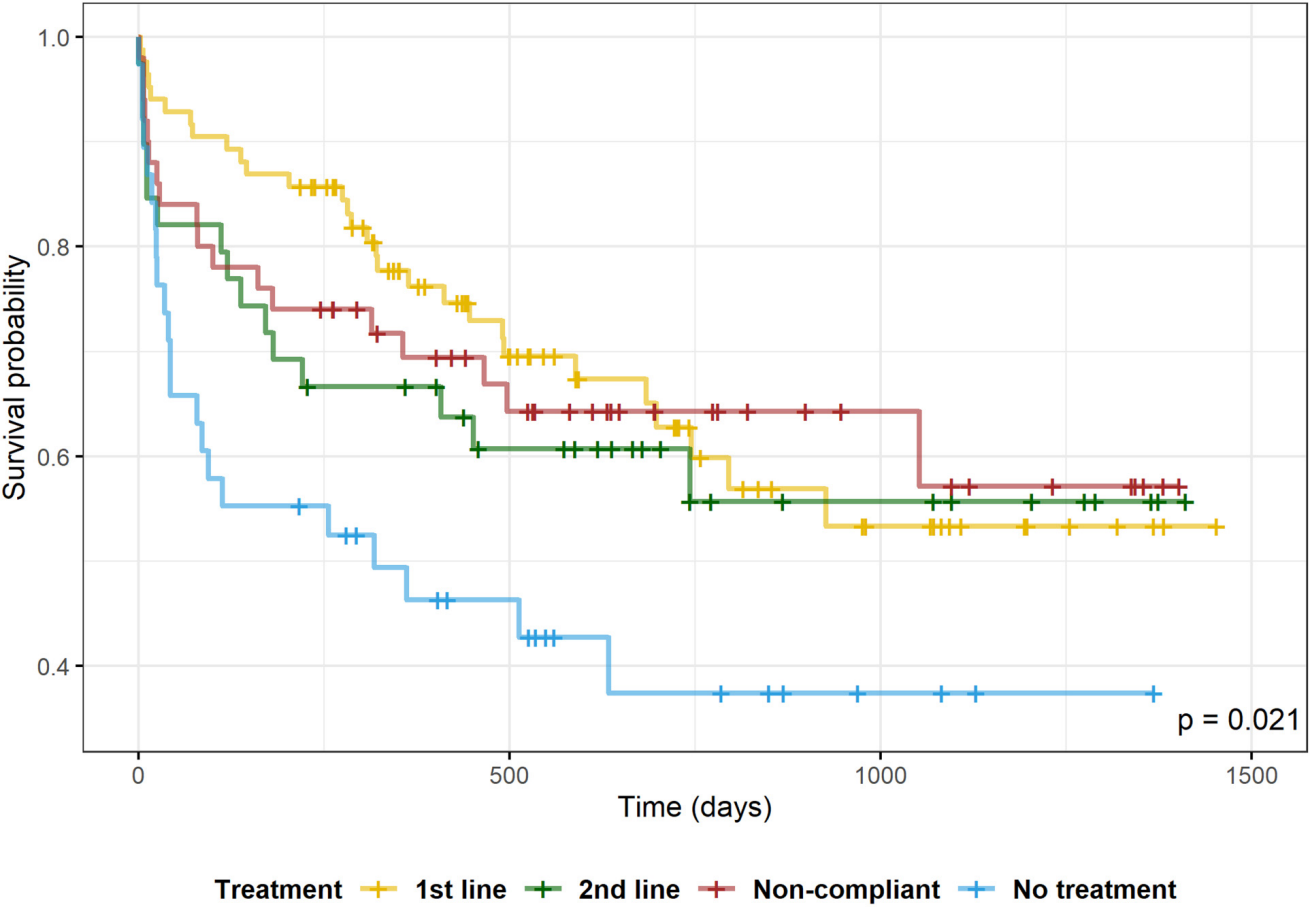
Kaplan–Meier survival analysis of time to hospitalization according to treatment classification. Log-rank test among survival curves (*p*  =  0.021); Log-rank test pairwise comparison between first-line treatment versus no treatment (*p*  =  0.014).

Mean time to rehospitalization was longer with first-line (mean  =  319.7 days; SD  =  268.2 days), second-line (mean  =  163.3 days; SD  =  208.7 days), and noncompliant regimens (mean+  = 188.3 days; SD  =  270.3 days) when compared to no treatment (124.3  ±  178.1 days). Figure 1 shows the results of the Kaplan-Meier survival analyses of the 4 groups (no treatment, first-line, second-line, and noncompliant regiments). We found a statistically significant difference among the survival curves (log-rank test, *p*  =  0.021) and pairwise comparisons showed a statistically significant difference between the curves of first-line and no treatment groups (log-rank test, *p*  =  0.014).

Patients who did not receive or refused treatment had a significantly increased risk of any readmission (hazard ratio=2.27; 95% CI, 1.30 to 3.96) compared to the group treated with first-line regimens. The multivariate Cox proportional hazards regression analysis showed that the patients that were on LTD support had a significantly increased risk of readmission (hazard ratio=2.52; 95% CI, 1.39 to 4.59). When controlling for LTD, the no-treatment group still presents an increased risk of readmissions (hazard ratio=2.34; 95% CI, 1.33 to 4.11). The interaction between the “no treatment” and “being on long-term disability support” variables was not statistically significant. Additionally, there was no significant statistical difference in the risk of rehospitalization when controlling for LAI.

## Discussion

In this retrospective cohort study of BD patients hospitalized for acute mania, we found that treatment with medications recommended as first-line options by the *CANMAT and ISBD 2018 Guidelines for the Management of Patients with Bipolar Disorder*^10^ was associated with lower rates of 30-day readmission and longer time to rehospitalization when compared to no treatment. Our results support the guideline rationale that medications that balance higher efficacy with better safety and tolerability profile and are classified as first-line treatments may be superior to other treatments in improving clinical outcomes such as short-term readmission rates and time to rehospitalization after an acute manic episode. It also suggests that the evidence from clinical trials which were used to rank the treatment options for acute mania is valid in a real-world context.

In addition to the effect of different treatments, we found an association between being on LTD and an increased risk of readmission during the follow-up period, despite no difference in the LTD rates according to treatment regimens at baseline. There was no statistically significant interaction between LTD and treatment groups in the survival analysis meaning that they are likely independent risk factors for any rehospitalization. This is in line with previous studies that showed that correlates of functional impairment and vulnerability such as homelessness, unemployment, and disability are associated with a greater risk of relapse and readmissions.^17–19^

Treatment with both first-line medications and noncompliant treatments decreased the risk of any rehospitalization when compared to no treatment during the study follow-up period. In our cohort, no patient received treatments classified as third-line, nonrecommended, or with recommendations against their use in acute mania (e.g. gabapentin, lamotrigine, or topiramate), and most patients classified as noncompliant with the guidelines were either on a combination of antipsychotics (e.g. LAI in combination with another second-generation antipsychotic or a combination of two second-generation antipsychotic) or using antipsychotics that are not listed as recommended treatments such as first-generation antipsychotics (e.g. loxapine and zuclopenthixol) and lurasidone. Antipsychotic polypharmacy is common in BD and although it may be as effective as standard treatments, it is usually not recommended due to its increased burden of side effects.^20^ Lurasidone use may also have contributed to the positive results of the noncompliant regimens in preventing any rehospitalization. Despite its efficacy in bipolar depression^21^ and schizophrenia^22^ and potential usefulness as an antimanic agent due to antipsychotic activity, there are no studies of lurasidone in mania and it is not listed as a recommended treatment by the guidelines. Unfortunately, due to the number of individuals and the large variability of medications in the noncompliant group, we were not able to explore the effect of each specific medication or combination on study outcomes.

In our sample, all patients classified as using second-line treatments were on olanzapine or a combination of olanzapine and a mood stabilizer, and its use was not associated with a statistically significant decrease in the risk of readmissions. Safety and tolerability issues were the main reasons why these effective treatments were reclassified as second-line options and our findings provide preliminary support for this decision by the CANMAT/ ISBD group.

The 2018 CANMAT and ISBD guidelines do not mention LAI antipsychotics as options for the treatment of acute mania. We decided to classify patients that were treated with LAI aripiprazole and LAI paliperidone as receiving first-line treatment based on the oral formulation recommendation and the fact that most patients initially receive oral and then transition to injectable medication. Even though LAI antipsychotics may be associated with more long-term compliance and lower hospitalization rates compared to oral formulations,^23,24^ there were no readmission rate differences in our cohort. Since we were not able to ascertain the continuity of LAI antipsychotics after discharge, it is possible that some patients did not remain on them, either by discontinuing the treatment or transitioning to oral formulations.

This study must be considered in light of its intrinsic limitations. We used a retrospective cohort design with few exclusion criteria, which allows for greater generalizability of results but is not able to control for important confounders due to its nonrandomized nature. We included patients with multiple admissions and although treatment groups were similar in terms of baseline features, we cannot rule out the influence of confounding by indication in our results. Multiple demographic and clinical data were extracted from the charts but other important variables such as a valid measure of the severity of symptoms and reliable information about continuation and/or adherence to medication after hospital discharge were not available. Our patients come from a single tertiary hospital and our findings must be replicated in larger samples and tested in prospective randomized studies.

Well-developed guidelines are a major advance in the treatment of complex illnesses such as BD, but their implementation remains a challenge. There have been increasing efforts from the CANMAT group to disseminate and implement the recommendations of the available guidelines. The *Patient and Family Guide to the CANMAT and ISBD Guidelines on the Management of Bipolar Disorder*^25^ was codeveloped with members from patient and advocacy groups to tailor the information to their needs and to encourage collaboration and shared decision-making. In the same vein, the *CANMAT Improving Patient Care and Outcomes in the Treatment of Bipolar Disorder (C-IMPACT BD) web-based application*^26^ was designed to improve clinician adherence to evidence-based pharmacological treatment recommendations based on point-of-care, patient-specific information. Based on preliminary data, the application increased the use of first-line treatments from 59.6% to 76.9%.^26^

Our results support the need for knowledge translation and implementation efforts of the *CANMAT and ISBD Guidelines on the Management of Bipolar Disorder* as we provide real-world data that first-line treatments, as a group, are associated with lower rates of short-term readmission after a hospitalization for a manic episode. Increasing physicians’ adherence to treatments with higher-ranked evidence for efficacy, safety, and tolerability may improve clinical outcomes in patients with BD.

## Supplemental Material

sj-docx-1-cpa-10.1177_07067437231156235 - Supplemental material for Does the Ranking Matter? A Retrospective Cohort Study Investigating the Impact of the *2018 CANMAT and ISBD Guidelines for the Management of Patients with Bipolar Disorder* Treatment Recommendations for Acute Mania on Rehospitalization Rates Click here for additional data file. Supplemental material, sj-docx-1-cpa-10.1177_07067437231156235 for Does the Ranking Matter? A Retrospective Cohort Study Investigating the Impact of the *2018 CANMAT and ISBD Guidelines for the Management of Patients with Bipolar Disorder* Treatment Recommendations for Acute Mania on Rehospitalization Rates by Fabiano A. Gomes, Henrique Dumay, Julia Fagen, Natalie Palma, Roumen Milev and Elisa Brietzke in The Canadian Journal of Psychiatry
