# Supplementary material for: Does the Ranking Matter? A Retrospective Cohort Study Investigating the Impact of the 2018 CANMAT and ISBD Guidelines for the Management of Patients with Bipolar Disorder Treatment Recommendations for Acute Mania on Rehospitalization Rates
Source: Can J Psychiatry. 2023 Feb 21;68(8):605–12. doi: 10.1177/07067437231156235 (PMC10411363; doi:10.1177/07067437231156235)
Supplement: sj-docx-1-cpa-10.1177_07067437231156235 - Supplemental material for Does the Ranking Matter? A Retrospective Cohort Study Investigating the Impact of the 2018 CANMAT and ISBD Guidelines for the Management of Patients with Bipolar Disorder Treatment Recommendations for Acute Mania on Rehospitalizatio [file sj-docx-1-cpa-10.1177_07067437231156235.docx]

**Supplementary Table 1:**

Demographic and clinical characteristics of the sample according to their treatment regimen (n=211)

|  |  | First line | Second line | Non-compliant | No treatment |  |
| --- | --- | --- | --- | --- | --- | --- |
| Events |  | 84 (39.8) | 39 (18.5) | 50 (23.7) | 38 (18.0) |  |
| Age, years (SD) |  | 42.54 (15.63) | 36.90 (12.95) | 41.00 (15.06) | 39.03 (13.39) | 0.93 |
| Gender | Male | 38 (18.01) | 25 (11.85) | 21 (9.95) | 16 (7.58) | 0.168 (f) |
|  | Female | 43 (20.38) | 14 (6.64) | 29 (13.74) | 22 (10.43) |  |
|  | Other | 3 (1.42) | 0 (0) | 0 (0) | 0 (0) |  |
| Marital status | Married | 25 (11.85) | 8 (3.79) | 14 (6.64) | 11 (5.21) | 0.75 |
|  | Not married | 59 (27.96) | 31 (14.69) | 36 (17.06) | 27 (12.80) |  |
| Living status | Independent | 79 (37.44) | 36 (17.06) | 48 (22.75) | 33 (15.64) | 0.40 (f) |
|  | Other | 5 (2.37) | 3 (1.42) | 2 (0.95) | 5 (2.37) |  |
| Long-term disability^a^ | Yes | 28 (13.53) | 12 (5.80) | 19 (9.18) | 16 (7.73) | 0.75 |
|  | No | 54 (26.09) | 26 (12.56) | 30 (14.49) | 22 (10.63) |  |
| Body mass index (BMI) | kg/m² (SD) | 26.24 (5.48) | 23.38 (5.23) | 24.72 (6.50) | 25.11 (6.59) | 0.98 |
| Family history of psychiatric disorders^b^ | Yes | 40 (20.51) | 19 (9.74) | 25 (12.82) | 17 (8.72) | 0.96 |
|  | No | 36 (18.46) | 19 (9.74) | 25 (12.82) | 14 (7.18) |  |
| General comorbidities | Yes | 58 (27.49) | 21 (9.95) | 37 (17.54) | 21 (9.95) | 0.11 |
|  | No | 26 (12.32) | 18 (8.53) | 13 (6.16) | 17 (8.06) |  |
| Psychiatric comorbidities | Yes | 72 (34.12) | 34 (16.11) | 39 (18.48) | 35 (16.59) | 0.32 (f) |
|  | No | 12 (5.69) | 5 (2.37) | 11 (5.21) | 3 (1.42) |  |
| Age of onset | Years (SD) | 29.79 (12.87) | 26.88 (9.06) | 28.19 (11.15) | 30.40 (14.58) | 0.97 |
| Previous suicide attempts^c^ | Yes | 17 (9.04) | 7 (3.72) | 10 (5.32) | 6 (3.19) | 0.86 |
|  | No | 59 (31.38) | 29 (15.43) | 31 (16.49) | 29 (15.43) |  |
| Length of hospital stay (index admission) | Days (SD) | 21.87 (20.70) | 14.90 (8.25) | 24.66 (25.23) | 8.26 (7.87) | 0.03 |
| Discharge type | AMA | 8 (3.79) | 5 (2.37) | 3 (1.42) | 9 (4.27) | 0.07 |
|  | Discharged | 76 (0.36) | 34 (16.11) | 47 (22.27) | 29 (13.74) |  |
| Long-acting injectable (LAI) medication | Yes | 32 (15.17) | 0 (0) | 9 (4.27) | 0 (0) | < 0.01 |
|  | No | 52 (24.64) | 39 (18.38) | 41 (19.43) | 38 (18.01) |  |

Legend: SD: standard deviation; AMA: against medical advice; (f) Fisher exact test; *Married also includes common-law and stable partner ^a^: Data unavailable for 04 patients; ^b^: Data unavailable for 16 patients; ^c^: Data unavailable for 23 patients
